# Digital Analysis of Smart Registration Methods for Magnetic Resonance Images in Public Healthcare

**DOI:** 10.3389/fpubh.2022.896967

**Published:** 2022-06-06

**Authors:** Tao Chen, Mengxue Yuan, Jiajie Tang, Long Lu

**Affiliations:** ^1^School of Information Technology, Shangqiu Normal University, Shangqiu, China; ^2^School of Information Management, Wuhan University, Wuhan, China; ^3^Institute of Pediatrics, Guangzhou Women and Children's Medical Center, Guangzhou Medical University, Guangzhou, China

**Keywords:** MRI, registration technology, feature comparison, SPM, FSL, AFNI

## Abstract

Brain development and atrophy accompany people's life. Brain development diseases, such as autism and Alzheimer's disease, affect a large part of the population. Analyzing brain development is very important in public healthcare, and image registration is essential in medical brain image analysis. Many previous studies investigate registration accuracy by the “ground truth” dataset, marker-based similarity calculation, and expert check to find the best registration algorithms. But the evaluation of image registration technology only at the accuracy level is not comprehensive. Here, we compare the performance of three publicly available registration techniques in brain magnetic resonance imaging (MRI) analysis based on some key features widely used in previous MRI studies for classification and detection tasks. According to the analysis results, SPM12 has a stable speed and success rate, and it always works as a guiding tool for newcomers to medical image analysis. It can preserve maximum contrast information, which will facilitate studies such as tumor diagnosis. FSL is a mature and widely applicable toolkit for users, with a relatively stable success rate and good performance. It has complete functions and its function-based integrated toolbox can meet the requirements of different researchers. AFNI is a flexible and complex tool that is more suitable for professional researchers. It retains most details in medical image analysis, which makes it useful in fine-grained analysis such as volume estimation. Our study provides a new idea for comparing registration tools, where tool selection strategy mainly depends on the research task in which the selected tool can leverage its unique advantages.

## Introduction

Image registration plays an essential role in image fusion, pattern recognition, and voxel-wise group analysis. Establishing correspondences across brains for comparison and group analysis is almost universally done by registering images to one another directly or *via* a template. Image registration is generally categorized into two groups, rigid and non-rigid registration. Rigid registration only consists of rotation and translation (1). Non-rigid registration is more complex and would take deformation methods, such as affine transformation and spline transform. There are a large number of optimized non-rigid registration algorithms and available registration tools, including advanced normalization tools (ANTS), analysis of functional neuroimages (AFNI), automated image registration (AIR), Drop, FMRIB's software library (FSL), and statistical parametric mapping (SPM). Thus, a comprehensive evaluation of different registration methods has become a research topic of interest. It is the basis for users to choose the most suitable methods for the specific research problem and for algorithm developers to be better informed theoretically (2). However, it is hard to assess image registration algorithms under different research contexts accurately. According to literature and survey, the main obstacles to building a unified evaluation standard of registration algorithm are as follows:

(1) No ground truth results are supplied in practice. In reality, the registration results are mainly assessed by an experienced expert. The lack of golden truth makes the evaluation research less convincing in terms of data. Also, it is hard to be flexible and straightforward to evaluate the performance of registration methods in a complex reality.
(2) Various parameter configurations for registration algorithms may lead to registered images of different quality. Experienced investigators can set appropriate parameters based on images' information. For the same input image, the difference in parameter setting would cause different registered outcomes (3).
(3) The same image data may be processed with different registration techniques to complete various tasks. Therefore, the data sets should be classified first and then preprocessed by other data registration methods according to different data characteristics.
(4) Data quality and raw data quality substantially impact registration results, which means that the data received by the registration program are quite different. The same algorithm may make a profound difference in the registration outcome when dealing with data with a significant quality difference.

Some researchers have done relevant research about the evaluation and comparison of registration algorithms to investigate this topic. Hellier et al. compared five different fully automated non-linear brain image registration software programs using the quantitative measures (4–6). Klein et al. evaluated 14 non-linear deformation algorithms applied to human brain MRI registration on four datasets and ranked these algorithms (7). Rajagopalan and Pioro investigated disparate voxel-based morphometry (VBM) results between SPM and FSL software in patients with ALS to determine which tool has the best performance of VBM in the ALS disease setting (8). Most recently, Dadar et al. compared publicly available linear MRI stereotaxic registration techniques by viewing the registered images by an expert rater to assess the quality of the registrations (9). At the same time, Viergever et al. made a retrospective view on the past two decades of medical image registration. They mentioned that “validation of registration methods and translation of image registration research results to the clinical practice still is the highlighted research and be more urgent than two decades ago (10).” Meanwhile, Rohlfing provides experimental evidence that registration accuracy measures such as tissue label overlap scores, image similarity, image difference, or transformation inverse consistency error, even when used in combination, cannot distinguish accurate from inaccurate registrations (11). Some previous accuracy standards are no more extended evaluation standards. Simulated data and a database with expert landmark annotations have been employed for comparison in the last decade to measure accuracy.

The registration of brain MRI with common templates is a long-standing problem. Different individuals' brain shapes and cortical topology are very different, especially in diseases affecting brain morphology and structure, such as brain atrophy. Previous researches mainly focus on registration accuracies, such as overlap, volume similarity, and registration error. However, the choice of registration technology has a more significant impact on image registration results. Few studies have used different registration tools to process image features. This means that the existing studies on the selection of image registration technology are insufficient. The image registration methods used in many studies are not standardized, and the accuracy level after registration is unknown and difficult to be quantified. Such problems will significantly affect the results of follow-up research. Therefore, the evaluation of image registration technology only at the accuracy level is not comprehensive. When facing different image processing projects, researchers need a flexible selection scheme to help them select the appropriate registration technology according to data sets' characteristics and personalized needs. Here, we investigate the performance of three publicly registration tools in two typical different disease cases, aiming to find a suitable registration tool for different disease types and research focus. Our research uses ABIDE and TCGA date sets to conduct experiments and deeply compares the registration performance of FSL, SPM12, and AFNI. In addition, we compare the three mainstream image registration technologies in terms of user experience and recommend them to different groups.

Our research investigates the registered results in two disease types, autism spectrum disorder (ASD), and glioma. Glioma may cause deformation in the morphology of the brain, and physicians can use high-resolution structural MRI images to detect the focus of infection and glioma grades. By contrast, ASD is connected with brain function and emotion and is often a pervasive developmental disorder. The pathogenesis of ASD is related to heredity, so the patient would have ASD at a very young age. The structural MRI and functional MRI images can be used to diagnose ASD in the clinic. We collect the MRI image data from public databases to construct our disease dataset. We do not intend to evaluate accuracy and error by manual labeling and check which are not available in reality. We plan to compare the features and characteristics of different tools' results because registration techniques are designed for follow-up analysis and application. We focus on the difference in registered results under various analyses, such as texture and edge-gradient features, voxel-based morphometry, and the impacts of this inter-method discrepancy on prediction and detection. From the application's perspective, we investigate the difference between three mature and powerful software to provide instructions for different researchers to select a suitable tool and more quickly get better achievements.

The structure of our study is as follows: in Materials and Methods, we describe the data acquisition and registration tools, then the strategy for data processing, and the ABIDE II and TCGA-LGG data set used to assess different registration tools. We focus on the difference in registered results under various analyses, such as texture and edge-gradient features, voxel-based morphometry, and the impacts of these inter-method discrepancies on prediction and detection. As a result, we instruct different researchers to select a suitable tool and more quickly get better achievements by investigating the difference between three mature and powerful software. Finally, in the discussion, we summarize the current work, emphasize the importance of selecting appropriate registration methods, and discuss the limitations and future improvement ideas.

## Materials and Methods

### Data Acquisition and Quality Control

This section introduces the basic information of ABIDE II-ETH and TCGA-LGG. Table 1 presents the basic information of the database.

(1) ABIDE II-ETH. Autism Brain Imaging Data Exchange II was established to further promote discovery science on the brain connectome in ASD (12). It is a multi-center and multi-scanner study involving 19 sites, ten charter institutions and seven new members, overall donating 1,114 datasets from 521 individuals with ASD and 593 controls (age range: 5–64 years). We select the ETH dataset to finish our research. There are 37 samples in the ETH set (age range 14–31 years), 13 for ASD and 24 for typical control (TC). Then, we manually check the ETH dataset to discard the bad quality image with serious artifacts.
(2) TCGA-LGG. The Cancer Genome Atlas Low-Grade Glioma (TCGA-LGG) data collection is part of a more significant effort to build a research community focused on connecting cancer phenotypes to genotypes by providing clinical images matched to subjects from The Cancer Genome Atlas (TCGA) (13). It contains CT and MRI data collected from 199 patients, and the number of studies in TCGA-LGG is 224. To gain the low-level glioma data with the same scan sequence and quality similar to the autism data set, we manually select a small part of the subject containing T2-w and T2-Flair sequence MRI data to finish our research.

**Table 1:** Dataset information.

**Dataset**	**ABIDE II-ETH**	**TCGA-LGG**
Slice thickness (mm)	0.9	2.5
No. of slices	180	69–80
Scan matrix	256 ×256	256 ×256
Repetition time (ms)	8.4	10,002
Echo time(ms)	22	125
No. of scans	31	27

### Registration Tools

#### SPM12

The SPM has several versions, and the latest is the SPM12 (14, 15). SPM12 is designed to work with MATLAB to run on Windows systems, while other similar tools are always supported by Linux or Mac and are easy to install. If someone is new to imaging, SPM12 is a recommended choice because plenty of tutorials give practical instructions on how to implement the various methodologies.

The spatial normalization method in SPM12 is a unified model based on a probabilistic approach. It combines the functions of image registration, tissue classification, and bias correction in the same generative model. The model is based on a Gaussian mixture and is extended to incorporate a smooth intensity variation and non-linear registration with tissue probability maps (15).

#### FSL

FSL is a software package developed by the Oxford Center for Functional MRI of the Brain (Oxford University), composed of image analysis and statistical tools for neuroimage data study. Flirt or Fnirt performed image registration in FSL. Flirt uses a multi-start, multiresolution global optimization method (16, 17), tailored explicitly for volumetric registration of brain images to give a reliable estimate of the worldwide minimum given some time restriction. The optimization algorithm uses four resolution levels: *n* = 8, 4, 2, 1 mm. Initially, the procedure starts with a large-scale search at 8 mm resolution (e.g., applying a set of initial rotations). Following this, various local optimizations are performed with multiple starting points in the local neighborhood of the best issues identified in the search. Then a series of multi-start local optimizations at 4 mm resolution is completed.

#### AFNI

AFNI is a leading software suite of C, Python, R programs, and shell scripts primarily developed to analyze and display anatomical and functional MRI (fMRI) data (18). The software is made to run on virtually a Unix system with X11 and Motif displays and is a widely used tool for image preprocessing. There are plenty of functions to perform registration, e.g., @auto_tlrc, 3dWarp, 3dWarpDrive, 3dAllineate, @toMNI_Awarp, @toMNI_Qwarp. The common function for registration is @auto_tlrc. This function is run by default parameters. Here, we use @auto_tlrc to execute registration (19).

#### Data Processing

We download data from the website and also check the quality and image label. After eliminating unqualified data, there are 33 for autism data sets (ASD + TC) and 45 for glioma data sets (glioma + TC) as a study data source. It is learned from Table 2 that the available data formats for each tool are not entirely the same. Firstly, we convert images of DICOM format into NIFTI-1 format. NIFTI-1 format is also the most commonly used international MRI format.

**Table 2:** Format requirements.

**Public tools data format**	**DICOM**	**ANALYZE**	**NIFTI_PAIR**	**NIfTI-1**	**NIFTI_GZ**	**HEAD/BRIK**	**MINC**	**CTF**	**ASCII**
SPM	√	√	√	√					
FSL		√	√	√	√				
AFNI	√	√	√	√	√	√	√	√	√

**Figure 1:**
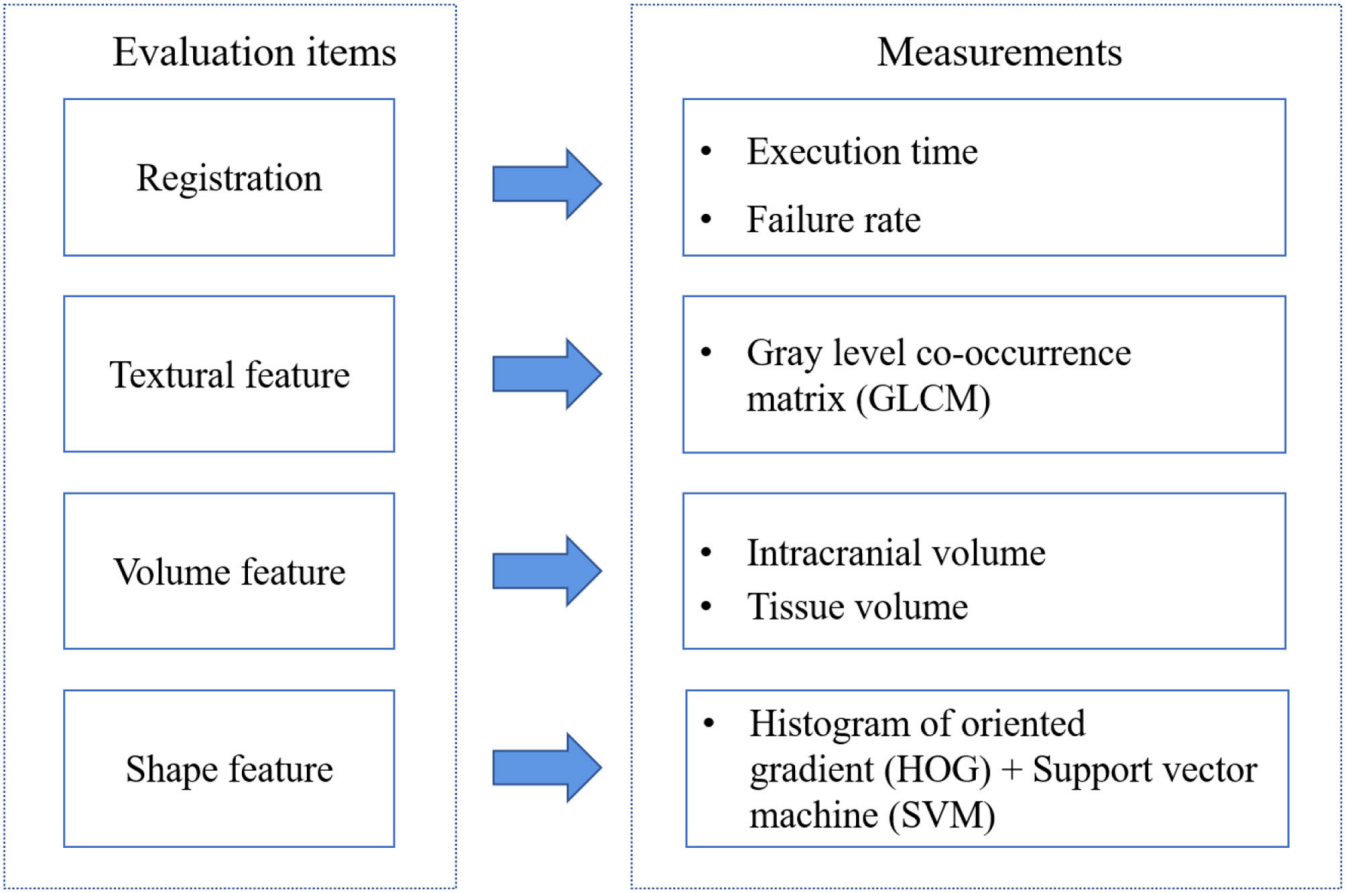
Comparison framework.

To explore the features difference among the three tools registered results, we construct the comparison framework shown in Figure 1. After organizing the dataset, we execute the registration step by AFNI, SPM, and FSL. T1-w and T2-FLAIR images were all registered to the standard space. SPM12 operations are finished on MATLAB R2012a. FSL and AFNI are installed and run in Ubuntu14 on a virtual machine.

There are three primary features for images: color, texture, and shape. Here, MRI images are gray images without color features. We consider volume as one of the evaluation items. Then we will introduce each item and its measurements.

##### Textural Feature

A textural feature is a diagnostic tool for physicians when reading MRI images. Learning from this, Zacharaki et al. extract MRI texture and shape features, constructing the feature vector to train a support vector machine (SVM) classifier to classify brain tumor type and grade (20). A textural feature is usually employed for tumor grading in tumor studies because it provides fine-grained, repeatable information. Besides, for autism, commonly diagnosed by behavioral, chemical, clinical, structural, and functional changes in the brain, MRI texture represents a new image feature, which can supplement the traditional image features (such as volume measurement). Vidhusha and Anandhan focused on extracting texture features for autistic and control subjects, and validated them using neural classifiers to achieve automatic detection of autism (21). Textural features are extracted from original MRI ROI images rather than registered results, but here, we explore the textural difference between results suffering different processing pipelines, and we will do a single statistical analysis to discuss the significant difference.

The textural feature has several extraction methods, including statistical, model, signal processing, and geometric forms. Here, we choose the gray level co-occurrence matrix (GLCM) proposed by Haralick et al. regarded as one of the most promising texture analysis methods (22, 23). It estimates the image properties related to second-order statistics. GLCM is a joint distribution describing the gray level of two pixels with a particular spatial positional relationship. Five derived variables were calculated from the GLCM matrix, contrast, correlation, energy, homogeneity, and IDM to investigate the difference between textural features. Then these computations will be described in detail next.

###### Contrast.

The deeper the texture groove, the greater the contrast and the clearer the visual effect; conversely, the contrast is small, the groove is shallow, and the effect is blurred.


(1) $$\begin{array}{r} contrast=\overset{N_{g}}{\underset{i=1}{\sum }}\overset{N_{g}}{\underset{j=1}{\sum }}{\left(i-j\right)}^{2}p\left(i,j\right) \end{array}$$

###### Correlation.

It represents the consistency of the image texture. If there is a horizontal direction texture in the image, the COR of the horizontal direction matrix is larger than the COR value of the remaining matrix.


(2) $$\begin{array}{r} correlation=\frac{\overset{N_{g}}{\underset{i=1}{\sum }}\overset{N_{g}}{\underset{j=1}{\sum }}p\left(i,j\right)ij-\mu_{x}\mu_{y}}{\sigma_{x}\left(i\right)\sigma_{y}\left(j\right)} \end{array}$$

###### Energy.

It is the sum of the squares of the gray level co-occurrence matrix values, so it is also called angular second moment, which reflects the uniformity of image gray distribution and texture thickness. A large energy value indicates a textured pattern that is more uniform and regular.


(3) $$\begin{array}{r} energy=\overset{N_{g}}{\underset{i=1}{\sum }}\overset{N_{g}}{\underset{j=1}{\sum }}{\left(p\left(i,j\right)\right)}^{2} \end{array}$$

###### Homogeneity.

It reflects the density of elements in GLCM relative to the diagonal distribution of GLCM.


(4) $$\begin{array}{r} homogeneity1=\overset{N_{g}}{\underset{i=1}{\sum }}\overset{N_{g}}{\underset{j=1}{\sum }}\frac{p\left(i,j\right)}{1+|i-j|} \end{array}$$

###### Inverse Difference Moment.

IDM measures the local homogeneity of an image and reports the inverse difference moment of an image. IDM weights are the inverse of contrast weights. It has the value that determines whether the print is textured or non-textured.


(5) $$\begin{array}{r} IDM=\overset{N_{g}-1}{\underset{k=0}{\sum }}\frac{p_{x-y}\left(k\right)}{1+k^{2}} \end{array}$$

##### Volume Feature

Volume could provide significant information on pathological study and can capture volume change in the brain. Quantitatively detecting the density and volume of brain tissue at the voxel level can reflect the differences in brain tissue components and characteristics in different brain regions. The latest study explores that the inter-method discrepancies in brain volume estimation may drive inconsistent findings in autism (3). In this part, we employ SPM12 as the brain volume estimation tool. The images are segmented into gray matter (GM), white matter (WM), and cerebrospinal fluid (CSF) volume in the native space to estimate the volume by using the New Segment tool of SPM12 (6). Spm_get_volumes script is used to calculate the tissue volumes using c1, c2, and c3 images corresponding to native space tissue maps of GM, WM, and CSF, respectively. Native space volumes are selected to minimize volume changes due to spatial transformations. Total intracranial volume (TIV) is calculated as the sum of the GM, WM, and CSF volumes in the native space of the structural MRI images.

##### Shape Feature

We calculate textural matrix to understand the textural feature and compare the brain tissue volume estimation, but it is hard to compare shape differences for shape features. Thus, we employ machine learning algorithms using shape features as the input and record the prediction accuracy to evaluate the shape feature performance. Based on the shape features extracted from MRI, machine learning algorithms could find the standard classifier function to distinguish the healthy and disease cases. To capture shape features in MRI images, we extracted the histogram of oriented gradient [HOG; (24)], a prominent local image feature widely used in computer vision applications, from MRI images to establish the feature sets. The HOG technique counts occurrences of gradient orientation in localized portions of an image. When studying the brain tumor, the HOG could describe local object appearance and shape within an image by distributing intensity gradients or edge directions. Thus, this descriptor can detect the abnormal edge of the tumor issues and help physicians find the lesions. About ASD, the HOG descriptor may also find some markers to distinguish the healthy and disease cases.

Then, the classification method is the support-vector machine (SVM), the supervised learning model. The combination of HOG and SVM is the classical strategy in object detection, especially in human detection (24). In the model, the cross-validation (CV) method is used to evaluate the performance of classifiers trained by different MRI data sets registered by three tools. The widely used methods in brain image analysis include leave-one-out cross-validation (25, 26), leave-two-out cross-validation (27–29), k-fold cross-validation (1), and stratified k-fold cross-validation (30, 31). The cross-validation results are conducted from the strategy that divides the raw data into K groups (K-fold), and each subset of data is used as a verification set. The remaining K-1 subset data is used as a training set. Then the K models, respectively, evaluate the results in the verification set, which effectively utilizes limited data, and the results are as close as possible to the model's performance on the test set. According to review studies and previous research, we use stratified ten-fold cross-validation to measure classification performance. All operations are performed in python3.7 in Ubuntu14 on a virtual machine.

## Results

### Time and Failure Rate

**Figure 2:**
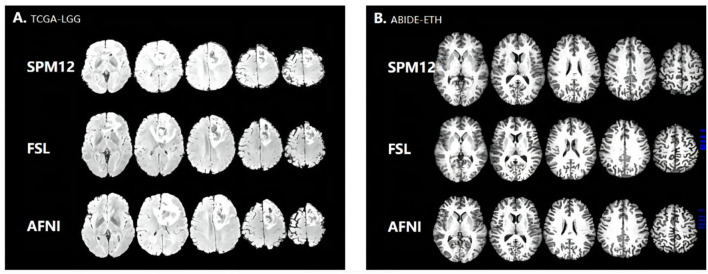
Registered results by three tools. **(A)** TCGA-LGG. **(B)** ABIDE-ETH.

Execution time is a crucial factor that needs to consider, which can reflect the effectiveness of tools. During the registration step, we calculate the time in each registration pipeline. Table 3 and Figure 2 show that AFNI gets the best speed performance and then SPM12 while FSL is the slowest.

**Table 3:** Time acquisition and failure rate.

	**ABIDE II-ETH**		**TCGA-LGG**	
	**Time (Average)**	**Failure rate (%)**	**Time (Average)**	**Failure rate (%)**
SPM12	94 s	0	100 s	0
FSL	110 s	0	4 min	16
AFNI	78 s	0	86 s	20

Registration is also regarded as an optimization process, which means there are failures in this step. We also collect the failure rate in the registration step. From Table 3, the failure rate in AFNI is higher than in FSL, and in FSL it is higher than in SPM. Auto-registration in SPM12 is based on prior knowledge using the tissue probability map to find the optimum transformation to achieve high and accurate registration. What is more, in SPM12, there is no feedback mechanism, and the final transformed results will be returned, whether good or bad. By contrast, FSL and AFNI both have the mechanism that if the cost function cannot be optimized, they will return the failure message and some additional suggestions to help the user optimize the registration command and get better results. So, AFNI and FSL have improvable abilities while SPM12 is poor.

The purpose of our study is not to attempt to find the most accurate one that has been investigated in many previous studies (7, 9, 32–34). We compare the registered results in the image to explore the degree of change in the features and structures made by the three registration tools and find the difference between these changes to help the user select the registration tool based on their registration results difference and research purpose.

### Texture Feature

We extract valuable features from the registered images by different tools to find the difference. Brain regions were extracted using the brain extraction tool [BET; (35)]. We extracted textural features from disease data sets to study the difference between registration tools and disease types (36, 37). Input images are brain-extracted MRI images to gain whole-brain texture GLCM matrix and calculate the five features, i.e., contrast, correlation, energy, homogeneity, and inverse difference moment (IDM).

**Figure 3:**
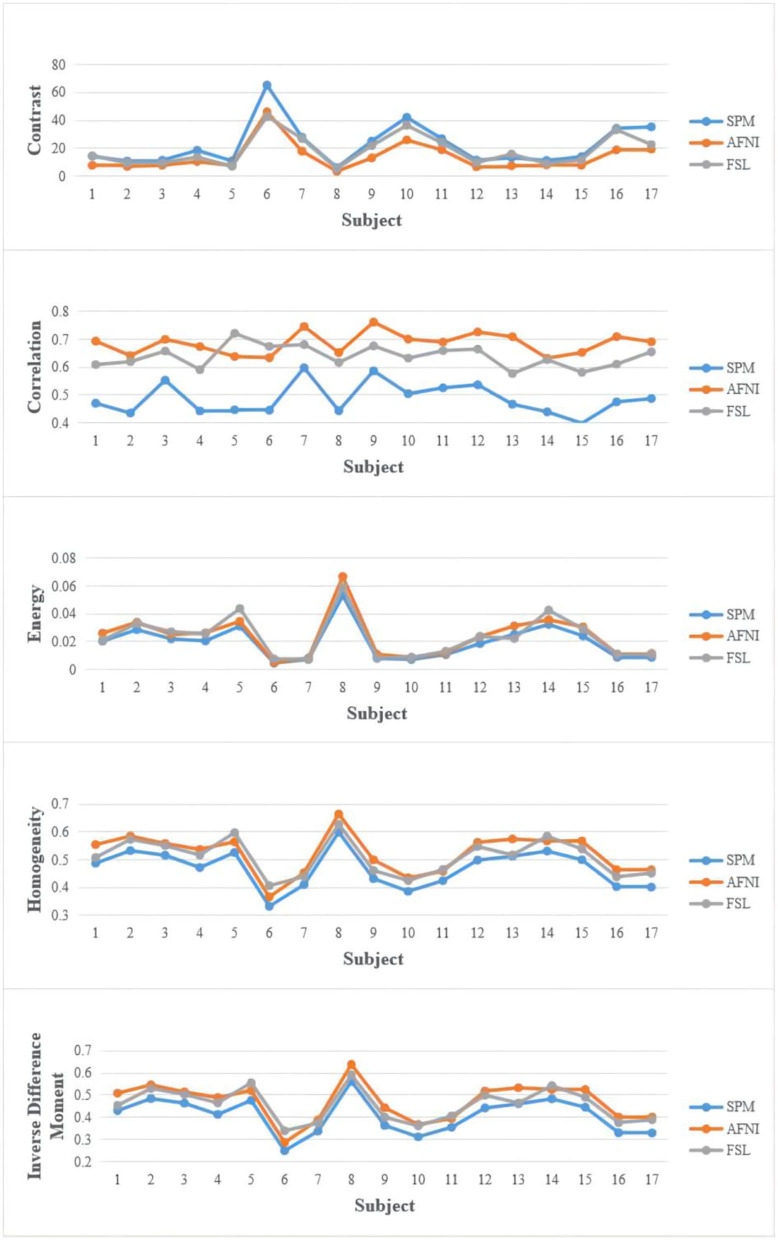
Texture feature extracted from the dataset.

Figure 3 shows the results of the comparison of texture features. The value distribution is similar among SPM, FSL, and AFNI and shows no significant difference. But FSL gets a higher value, implying that FSL is more inclined to retain more uniform and regular texture patterns. AFNI has a higher correlation value corresponding to its biggest size of MRI holding more pixels and details that could be measured more similar pattern.

In the statistical analysis, SPM12 values shows significant difference with AFNI (*p* = 0.017314065 < 0.05, 95%) and FSL (*p* = 0.046368617 < 0.05, 95%), indicating that SPM12 registered images have more sharp textural changes. A larger contrast value correlates with a greater disparity in intensity values among neighboring voxels, suggesting that SPM12 processed results have more sharp intensity changes and deeper texture grooves. Next, the inverse difference moment, also known as the inverse variance, reflects the clarity and regularity of texture. The images with clear, regular, easy to describe texture would have a larger value demonstrating that these images are more in line with human feelings. The AFNI is clearer than the other two data sets in Figure 2, which is consistent with our calculated values. SPM12 also has significant difference with AFNI (*p* = 0.018590934 < 0.05, 95%) and FSL (*p* = 0.044412676 < 0.05, 95%) as well. We guess the reason is that AFNI retains more detailed information, including uniform and sharp textural change, while SPM12 abandons these details and hold sharp textural feature.

### Brain Volume Estimation

**Figure 4:**
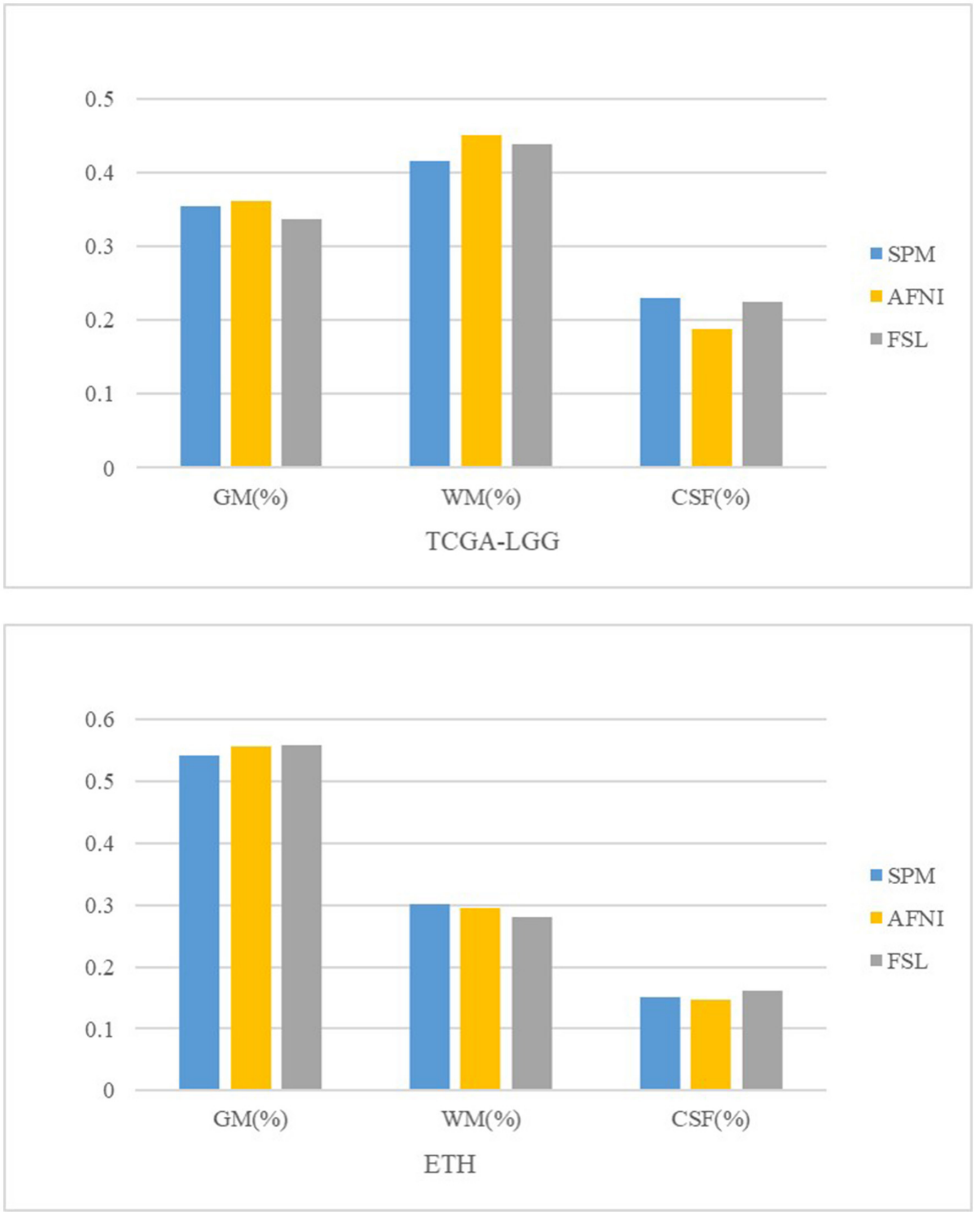
Brain tissue volume comparison.

Some brain diseases may give rise to the volume change in the brain, such as edema, which is obvious in the image. We calculate the TIV and the percentage of gray matter, white matter, and CSF in the original and registered structural MRI images. We set the volume estimation from the MRI in the original space as the standard result. From Table 4 and Figure 4, AFNI shows the closest results with similar GM, WM, and CSF percentages, and sometimes overestimates the total volume. The whole-brain volume estimated by SPM was always higher than the standard TIV. FSL estimates of CSF are the highest.

**Table 4:** Brain volume estimation results.

	**TIV (mean)**	**GM% (mean)**	**WM% (mean)**	**CSF% (mean)**
TCGA-LGG	1.5364	39.89	45.17	14.94
SPM (TCGA)	1.9012	35.41	41.61	22.97
AFNI (TCGA)	1.8936	36.08	45.13	18.79
FSL (TCGA)	1.5123	33.62	43.89	22.49
ETH	1.2960	58.79	32	9.21
SPM (ETH)	1.8776	54.21	30.17	15.02
AFNI (ETH)	1.8977	55.63	29.63	14.74
FSL (ETH)	1.5641	55.81	28.01	16.19

*ETH = ABIDE II-ETH*.

### Shape Feature-Based Classifier

Finally, we compare the evaluated accuracy of the cross-validation. For the ASD dataset, the number of healthy and disease cases is 22 and 11, respectively. For the glioma dataset, the number of healthy and disease cases is 22 and 17, respectively, and the k that we use in k-fold is 10.

From Table 5, we can observe the difference between the registration tools. The performance of SPM shows the highest accuracy, while AFNI is better than FSL. Since the HOG feature represents the shape information by edge directions, which collects contrast information, this result is consistent to compare results found in the texture feature part. MRI images registered by SPM retain richer contrast sources, which may mean that SPM is a suitable preprocessing tool when dealing with structural problems. The SPM performs best in our structural HOG feature model.

**Table 5:** Prediction results on two datasets.

**ABIDE II-ETH dataset**	**SPM %**	**AFNI %**	**FSL %**
Accuracy (high)	78.79	72.73	63.64
Accuracy (low)	69.70	63.64	54.55
**TCGA-LGG dataset**	**SPM %**	**AFNI %**	**FSL %**
Accuracy (high)	92.31	88.83	81.17
Accuracy (low)	86.24	80.52	72.08

## Discussion

To estimate the performance of registration algorithms of SPM, FSL, and AFNI, we calculate the registration time, compare the texture feature extracted from skull-striped structural MRI images, and investigate the inter-methods discrepancies in brain volume estimate. Finally, we compare these registered images' applied performance in the classification model and we get the conclusion that SPM12 is usable for newcomers or employed when dealing with basic and normal processing workflow because it is like a black box that users cannot know the registration process details. FSL is a powerful toolkit for research. Its function-based integrated toolbox facilitates researchers for the different hierarchy of requirements. However, FSL's semi-integrated design cannot meet scholars' requirements for function module combinations and workflow model optimization. In contrast, AFNI is cumbersome and complex to use. However, it can provide some personalized customization and allow some threshold adjustments, which is very useful for many professional researchers, but it may be difficult for novices to use.

According to our analysis conclusion, tool selection mainly depends on research tasks. If the focus of studies is only MRI images' single feature, it is wise to choose the feature-targeted tools, for example, the contrast feature of texture feature for SPM, and if studies require multiple features or detailed features, AFNI will be a good choice. When involving multi-modal MRI sequences, like DWT, PET, and DTI, FSL would become the first choice. For glioma and autism spectrum disorder, the glioma is more sensitive to contrast features, so SPM12 may perform better registration results when studying the glioma dataset. When using the shape features like HOG, SIFT, or LBA, SPM12 may have the best performance on comparing AFNI and FSL. Tumor detection, prediction, and classification are likely to employ these structural and edge-related features, and so SPM12 will be an advisable process tool with stable performance and feature-sensitive advantages, facilitating tumor study.

Image registration research is still progressing steadily, and new research results are emerging. At present, there is still no unified evaluation criterion. Experience and habits are still the main factors affecting people's choice of methods and tools. Our study provides a new idea for the comparison of registration tools. Using data sets from different diseases, we study the differences in texture features, structure, and classification performance of the three registration tools, discuss their characteristics, and analyze the causal correlation between features and classification models. Our study is not without limitations. The focus of this study is only on structural images, while the registration between functional images and other modal data has not been investigated in detail. Secondly, only three kinds of public registration tools are selected, and some tools in current research are omitted. In addition, the default parameter settings are adopted in the study. Still, in the specific analysis, different parameter settings will significantly impact the results, and the appropriate parameter configuration can optimize the processing results. There is some research on MRI registration using a deep learning algorithm, which may achieve better performance than traditional MRI registration methods. In the next step, we need to compare and analyze the registration results of these methods (38, 39).

## Conclusions

Although SPM has the most stable performance, it also produces poor registered results when dealing with low-quality data sets. According to its advantages and drawbacks, SPM12 has a stable speed and success rate but does not guarantee the processed results, especially when handling low-quality images. FSL is a mature and widely applicable toolkit for employers, with relatively stable performance, a success rate, and complete functions. Its graphical user interface provides basic processing flow operation, while multi-parameter settings and failure feedback mechanisms can realize complex user processing and meet the optimization requirements. It is an excellent toolbox for beginners and proficient hands. Last, AFNI, with quite a fast speed and complex modules, can meet various requirements. But it is not friendly to newcomers, even though it offers an integrated processing function-similar function and modules, unique data format, and command-line operation, all confusing to beginners.

The texture feature is significant in image registration. SPM registration enhances the contrast feature of the texture feature, giving up other detailed information. FSL registration contains enough contrast information and most of the details. AFNI registration is apparent to view details in the brain structure. Due to plentiful details, too much information may make finding key features to differentiate brain structure by single textual features. Therefore, SPM registered image is easy to distinguish the different and distinct tissue types in the images. MRI images processed by AFNI and FSL are more suitable for complex and fine-grained investigations.

In conclusion, AFNI will be a good product application for its immediate and quick response with processed results but requires extra steps to ensure quality. It also plays an essential role in academics and research with its excellent flexibility, which could quickly test various combined processing steps in research. It is also the only MRI registration tool that supports all formats. SPM12 always works as a guide tool for newcomers to medical imaging. A rich online teaching resource and simple and easy-to-learn operations make everyone accomplish the basic processing flow and get stable results. Its support for the Windows environment is the main reason for its popularity. FSL combined advantages of both, providing GUI for basic and simple processing flow and customized parameters setting for flexible and optimized procedure. In addition, FSL is a suitable tool for other MRI sequences like DWT and has been widely used in numerous studies.

## Data Availability Statement

Publicly available datasets were analyzed in this study. This data can be found at: https://www.nitrc.org/frs/downloadlink.php/9090 ABIDE II-ETH (39); https://wiki.cancerimagingarchive.net/display/Public/TCGA-LGG TCGA-LGG (224).

## Author Contributions

All authors listed have made a substantial, direct, and intellectual contribution to the work and approved it for publication.

## Funding

This research was funded by National Natural Science Foundation of China (61772375, 61936013, and 71921002), the National Social Science Fund of China (18ZDA325), National Key R&D Program of China (2019YFC0120003), Natural Science Foundation of Hubei Province of China (2019CFA025), Independent Research Project of School of Information Management of Wuhan University (413100032), the Key R&D and Promotion Projects of Henan Province of China (212102210521), and the Key Scientific Research Project in Colleges and Universities of Henan Province of China (22A520038).

## Conflict of Interest

The authors declare that the research was conducted in the absence of any commercial or financial relationships that could be construed as a potential conflict of interest.

## Publisher's Note

All claims expressed in this article are solely those of the authors and do not necessarily represent those of their affiliated organizations, or those of the publisher, the editors and the reviewers. Any product that may be evaluated in this article, or claim that may be made by its manufacturer, is not guaranteed or endorsed by the publisher.
